# Anterior Segment Optical Coherence Tomography with Angiography for the Cornea and Ocular Surface

**DOI:** 10.3390/jcm15062402

**Published:** 2026-03-21

**Authors:** Qiu Ying Wong, Ralene Sim, Marcus Ang

**Affiliations:** 1 Singapore Eye Research Institute, Singapore National Eye Centre, Singapore 168751, Singapore; wong.qiu.ying@seri.com.sg (Q.Y.W.); ralene_sim1995@hotmail.com (R.S.); 2 Ophthalmology and Visual Sciences Academic Clinical Program, Duke-NUS Medical School, Singapore 169857, Singapore

**Keywords:** anterior segment, optical coherence tomography, optical coherence tomography angiography, ocular surface disease

## Abstract

**Background/Objectives**: Anterior segment optical coherence tomography (AS-OCT) and optical coherence tomography angiography (AS-OCTA) have enhanced the evaluation of the cornea, ocular surface, and ocular surface diseases (OSD), offering high-resolution structural and anterior segment vascular imaging. This review summarizes recent advances in these modalities and their clinical applications. **Methods**: A comprehensive literature search was conducted using PubMed, Web of Science, and Google Scholar with the terms OCT, OCTA, anterior segment, and ocular surface disease. Studies published in the past five years were included, emphasizing more recent developments such as ultra-high-resolution AS-OCT (UHR-AS-OCT) and swept-source AS-OCTA technologies. **Results**: UHR-AS-OCT provides non-invasive, sub-micron imaging of the cornea and the ocular surface, including tear film morphology and epithelial thickness to correlate with clinical tests such as tear break-up time, and fluorescein staining. Advances in AS-OCTA allow dye-free, depth-resolved imaging of corneal and conjunctival vasculature. These vascular biomarkers have shown promising utility in conditions such as limbal stem cell deficiency, chemical ocular injury, and ocular surface squamous neoplasia. Improvements in image acquisition, motion correction, and segmentation algorithms have enhanced accuracy and repeatability, supporting broader clinical translation. **Conclusions:** AS-OCT and AS-OCTA have become useful adjunctive imaging tools for the cornea and ocular surface evaluation. Their non-invasive, quantitative, and reproducible metrics may enable earlier diagnosis, objective staging, and longitudinal monitoring of OSD. Integration of OCT-based imaging with artificial intelligence and multimodal data, including tear proteomics and meibography, may optimize personalized treatment for ocular surface disorders.

## 1. Introduction

Optical coherence tomography (OCT) in the anterior segment of the eye has undergone substantial technological advancements since its introduction, with applications pioneered by Izatt et al. in 1994 [1]. As a non-invasive imaging modality, it generates three-dimensional tomographic images by using low-coherence light, enabling high-resolution, in vivo visualization of ocular structures [2,3]. Recent advances have led to the development of ultra-high-resolution anterior segment OCT (UHR-AS-OCT), achieving axial resolutions of less than 5 μm [4]. This enables better precise measurements and reveals details of the cornea and ocular surface that may not usually be discernible with conventional OCT platforms. However, evaluating ocular surface diseases (OSD) requires holistic assessment of the eye and its adnexal components, such as the cornea, conjunctiva, tear film, eyelids, eyelashes, lacrimal glands, and meibomian glands [5].

Fluorescein angiography (FA) and indocyanine green angiography (ICGA) remain the gold standards for evaluating vascular leakage and perfusion, yet their invasive nature, time-intensive protocols, and risk of adverse effects have underscored the need for advanced non-invasive imaging alternatives [6]. Optical coherence tomography angiography (OCTA) allows rapid, non-invasive acquisition of high-resolution angiographic images of the eye across multiple en face planes within seconds [7]. Since its commercial introduction in 2014, OCTA has enabled the delineation of blood vessels through motion contrast imaging by detecting phase and speckle contrast, as well as intensity variations derived from consecutive B-scans [8,9]. OCTA is well established for imaging retinal microvasculature and detecting pathologies such as retinal neovascularization, with recent adaptations extending its use to the anterior segment for detailed visualization of corneal and conjunctival vessels [10].

Although initially constrained by a narrow field of view, motion artifacts, and interpretative challenges, recent advances such as swept-source anterior segment OCTA (AS-OCTA) allow faster acquisition, wider imaging fields, and improved image processing, enabling accurate, comprehensive visualization of corneal and conjunctival vessels and expanding its clinical applications to OSD including corneal neovascularization, pterygium, and surface tumors [11,12]. Despite these advances, clinical integration remains limited by small patient cohorts and lack of standardized imaging protocols.

Recent reviews have summarized the applications of AS-OCTA in imaging the cornea and ocular surface [2,13,14]. Lee et al. provided a comprehensive overview of AS-OCTA technology and its early applications in evaluating corneal and conjunctival vascular structures [15]. However, since that publication, there has been increasing clinical interest in integrating OCT and OCTA imaging into the diagnosis, monitoring, and management of OSD.

### What Is New in This Review

The present review extends prior work by providing a broader synthesis of both structural OCT and OCTA applications in ocular surface diseases. In addition to summarizing imaging findings, this review focuses on the clinical utility of OCT-based biomarkers, longitudinal monitoring of disease activity, and therapeutic response assessment, areas that have gained attention in the recent literature. We also discuss important limitations of current OCTA metrics, including measurement variability and the need for validated quantitative thresholds for clinical interpretation. By integrating structural and vascular imaging advances, this review aims to provide an updated perspective on the evolving role of OCT-based imaging in ocular surface disease research and clinical practice.

## 2. Methodology

This article was conducted as a structured narrative review aligned with SANRA (Scale for the Assessment of Narrative Review Articles) principles to ensure clarity of scope, transparent literature identification, and critical synthesis [16]. A targeted literature search was performed using PubMed/MEDLINE and Embase for studies published between years 2020 and 2025. Search terms included combinations of: “anterior segment OCT,” “ultra-high-resolution OCT,” “AS-OCTA,” “epithelial thickness mapping,” “tear film thickness,” “limbal stem cell deficiency,” “corneal neovascularization,” “quantitative imaging,” and “reproducibility.” All retrieved records were exported into reference management software, and duplicates were identified and removed prior to screening. Reference lists of key articles were manually screened to identify additional relevant publications. Screening was performed by the authors, with disagreements resolved through discussion. The initial search identified approximately 1247 records. After duplicate removal (*n* = 260), 987 unique records were screened by title and abstract. Of these, 489 full-text articles were assessed for eligibility and selected work pertinent to the topic were included in this review.

Studies were considered eligible if they reported original human research involving AS-OCT or AS-OCTA and presented quantitative imaging metrics relevant to anterior segment structure or vascular assessment. Emphasis was placed on studies reporting epithelial thickness mapping, tear film thickness measurements, vascular density or flow metrics, and reproducibility indices such as intraclass correlation coefficients (ICC). Studies evaluating diagnostic performance, clinical correlations, or longitudinal reliability were prioritized. Only peer-reviewed articles published in English were included. Studies were excluded if they consisted of single case reports or small case series involving fewer than five participants, focused exclusively on posterior segment OCT imaging, or did not report quantitative imaging parameters. Purely technical engineering reports without clinical application were excluded, as were conference abstracts without full peer-reviewed publication. Review articles were not included in the synthesis, although their reference lists were screened to identify additional relevant primary studies.

Given the narrative design, formal inclusion/exclusion criteria, risk-of-bias assessment, and meta-analysis were not performed. However, methodological considerations, including sample size, device heterogeneity, reproducibility metrics (ICC), and study design, were critically appraised and incorporated into the interpretation of findings. Emphasis was placed on studies reporting effect sizes, correlation magnitudes, and reproducibility indices to contextualize clinical relevance. In view of substantial heterogeneity across imaging platforms, scan protocols, and segmentation algorithms, quantitative findings are presented descriptively rather than as directly comparable benchmarks. Where available, effect sizes, correlation magnitudes, and reproducibility metrics are reported to contextualize technical performance within clinical relevance. Given rapid technological evolution and cross-platform variability, this review focuses on representative studies illustrating key quantitative concepts rather than providing comprehensive comparative device evaluation. Table 1 outlines evidence based on recent applications of OCT and OCTA in the anterior segment incorporated into this review.

## 3. Early-Stage Ocular Surface Disease: Insights from OCT

### 3.1. Tear Film Analysis

The precorneal tear film (PCTF) is pivotal for preserving ocular surface homeostasis, creating a smooth air–tear interface, and serving as a protective barrier against infection and desiccation [18]. Quantification of the tear film thickness (TFT) has been investigated using optical approaches, which include confocal microscopy and interferometry, but reported substantial differences in values ranging from 3 μm to 46 μm [26,27]. Factors affecting the integrity of the lacrimal film can vary externally, including environmental conditions, interval between blinks, and palpebral aperture dimensions, contributing to its multifactorial nature in dry eye disease (DED) [28].

**Figure 1:**
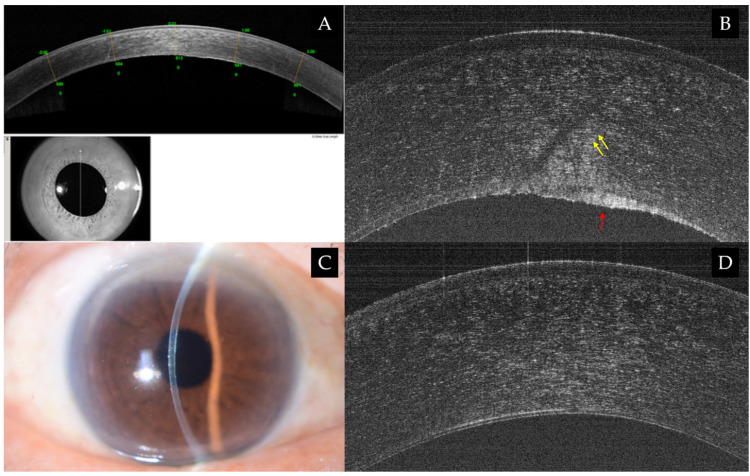
(**A**–**C**) Clinical images of an eye with superior stromal edema (yellow arrows) secondary to Fuchs Endothelial Corneal Dystrophy (FECD) compared to a healthy cornea (**D**). No obvious focal thickening or thinning of the corneal structure captured on slit lamp photo (**B**) or cross-sectional Bscan (RTVue-100; Optovue Inc., Fremont, CA, USA) (**A**). Enhanced axial resolution (<2 μm) by UHR-AS-OCT of 800 nm wavelength using a superluminescent diode revealed fine anatomical details illustrating areas of focal thickening (red arrow) in the FECD-affected cornea relative to the healthy cornea (**D**). Its superior resolution facilitates reliable differentiation between pterygium and ocular surface malignancy, characterized by features, i.e., epithelial thickening, increased hyper-reflectivity, and well-defined transition zones [38].

Axial resolution of commercially available AS-OCT systems ranges from 2 to 25 μm, whereas custom-built UHR-AS-OCT offers resolution ranging from under 5 μm down to an order of 1 μm, facilitating more meticulous scanning of anterior segment ocular structures [17,29,30] as shown in Figure 1. Of which, TFT in normal eyes yielded an estimated value of 3 μm and have been quantified reproducibly (4.79 ± 0.88 μm) through a spectrometer-based UHR-AS-OCT (ICC: 0.97) [31,32]. The discrepancy between DED patient-reported outcomes and clinical findings persists as a key limitation of current diagnostic techniques [33]. Accordingly, TFT measured by UHR-AS-OCT, using sapphire laser with a resultant theoretical axial resolution of 1.2 μm demonstrated signification correlation with Ocular Surface Disease Index (OSDI) (*r* = −0.34, *p* = 0.01) in this pilot study of 52 patients with DED. While low TFT may particularly be found in patients with aqueous deficient DED, TFT may vary in other natures of DED (e.g., meibomian gland disease-related, evaporative) [34,35]. Although evidence for using TFT to evaluate therapy or different forms of OSD is still lacking, studies have reported increases in TFT for up to 24 h with topical agents such as low-dose hydrocortisone and perfluorohexyloctane [36,37].

The lipid layer, a key constituent of the tear film, acts as a surfactant to reduce surface tension and maintain a smooth air–tear surface. However, these functions are impaired in meibomian gland dysfunction, as any obstruction or alterations in the meibum can reduce the quality and quantity of the supply of lipids to the tear film lipid layer (TFLL) [39]. This disruption accelerates tear film evaporation and compromises its stability, thereby contributing to the development of evaporative DED. Quantification of the TFLL in vivo remains a fundamental constraint due to its ultra-thin nature. Nevertheless, volumetric data from AS-OCT can be projected axially to create en face maps, achieving in vivo visualization of the TFLL [40]. Stegmann et al. recently proposed a new classification model for TFLL en face maps using UHR-AS-OCT and its correlation with objective clinical parameters such as fluorescein breakup time (FBUT) and Schirmer test [18,41]. Qualitative TFLL patterns—homogeneous (HOM), wavy (WAV), and dotted (DOT)—were identified, with the DOT pattern (characterized by a dark background with bright spots) indicating the thickest average TFLL and highest FBUT, suggesting that it may represent the most stable form of the lipid layer [18]. Furthermore, the evaluation of PCTF and TFLL has been suggested with a known interaction of the latter’s function in reducing evaporation of the PCTF, potentially revealing useful insights in the structure–function relationship of the tear film and lipid layer in MGD (meibomian gland dysfunction). Bai et al. reported that while PCTF thicknesses were similar across normal and MGD (asymptomatic and symptomatic) groups, the MGD eyes exhibited a more rapid rate of PCTF thinning than the controls, presumably reflecting a compensatory increase in aqueous tear production [19].

### 3.2. Dry Eye Disease

Maintaining ocular integrity relies heavily on the ocular surface, which functions as a defensive barrier to external factors. Ocular surface disease (OSD) then arises from the disruption of these mechanisms that preserve the ocular surface integrity, encompassing a wide spectrum of disorders with varied etiologies [42]. Dry Eye Disease (DED) is a multi-factorial disease that is characterized by a “loss of homeostasis of the tear film, and accompanied by ocular symptoms, in which tear film instability and hyperosmolarity, ocular surface inflammation and damage, and neurosensory abnormalities play etiological roles [5].” The complexity of DED stems from inflammation in many domains and is not limited to autoimmune-based (e.g., Sjögren’s Syndrome, Stevens–Johnson Syndrome) and non-immune-based (e.g., meibomian glands disease, corneal transplantation), leading to OSD. Current objective tests (e.g., FBUT and fluorescein dye staining) used to evaluate tear film stability remain controversial, with the latter potentially leading to false conclusions due to induced discomfort and reflex tearing [43].

AS-OCT has become a valuable non-invasive modality for the quantitative evaluation of tear film morphology and dynamics. Czajkowski et al. demonstrated the strong correlation between tear meniscus parameters—namely tear meniscus area (TMA)—tear meniscus height (TMH), and tear meniscus depth (TMD) with spearman correlation coefficient values as 0.54, 0.52, and 0.3, respectively. For example, TMA and TMH measured by AS-OCT had significant correlation with subjective measures (e.g., OSDI) and reported symptoms, suggesting good sensitivity and specificity in diagnosing DED (86.11% and 85.33% using TMA and 80.56% and 89.33% using TMH) [44]. Popovici and Banc, however, observed no significant differences between DED and controls using similar AS-OCT parameters (e.g., TMH, TMA, TFT) when using OSDI and examination confirmation as a diagnostic tool [45].

Recent work with swept-source AS-OCT (Casia2, Tomey, Nagoya, Japan) across diverse OSD cohorts, including Sjögren’s Syndrome and corneal transplant patients, demonstrated good repeatability with other anterior segment parameters (e.g., anterior chamber depth, width, and angle) with no significant differences (*p* > 0.05), suggesting the overall measurement consistency of AS-OCT [46]. Shousha et al. demonstrated that their novel UHR-AS-OCT, with a 3 μm axial resolution, can generate corneal epithelial profile (CEP) maps capable of detecting microscopic epithelial irregularities, quantified as the epithelial irregularity factor (EIF). EIF values were significantly higher in DED compared with controls (5.79 vs. 0.77, *p* < 0.001) and showed a strong correlation with patient-reported symptoms (*r* = 0.778, *p* < 0.001). This technique may therefore serve as a useful tool for monitoring disease progression and treatment response [17].

### 3.3. Cornea Epithelial Thickness Mapping

**Figure 2:**
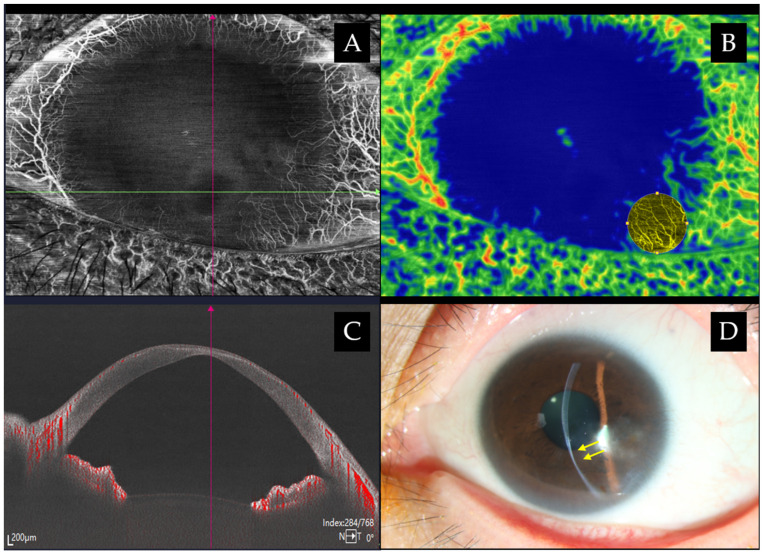
Clinical images of an eye with localized corneal thinning and scarring due to previous infection. (**A**) An en face image with 360° pannus and superficial vascularization inferotemporally using swept-source AS-OCTA (BMizar, BM-400K, TowardPi Medical, Beijing, China). (**B**) Corneal neovascularization quantification maps can be used to enhance areas of interest with CoNV lesion demarcated in yellow. (**C**) Cross-sectional B scan illustrating areas of blood flow signals. (**D**) Slit-lamp photo with beam slit narrowing towards area of thinning (yellow arrows).

The corneal epithelium constitutes approximately 1.03 D of the total corneal refractive power within the central 2 mm zone and about 0.85 D within the 3.6 mm zone, with the slightest modification to the epithelial thickness (ET) and morphology can trigger substantial refractive changes [47]. The mechanical forces exerted by the eyelid and blinking action superiorly are presumed to contribute to its nonuniform thickness, with the cornea being thickest inferiorly, followed by the superior and nasal regions, and the thinnest temporally [48,49]. AS-OCT has demonstrated repeatable, reproducible epithelial thickness mapping (ETM) in both normal and diseased cornea, as illustrated in Figure 2, and explored in dry eye patients and animal models [50,51]. Although in vivo confocal microscopy (IVCM) allows the precise measurement of ET, defined as the distance between superficial and basal corneal epithelial cells, its relatively invasive nature limits widespread clinical applicability. Shortcomings of Spectral Domain-OCT (SD-OCT) technology have been noted as the overestimation of the tear film due to the lack of resolution [28].

With the advent of newer AS-OCT systems, a larger area is imaged on corneal ET maps. A total of seventeen zones over a 6 mm diameter is covered, namely the central 2 mm diameter, eight 3 mm paracentral and eight 1 mm peripheral zones [52]. Several parameters can be derived from ETM—namely, minimum corneal thickness (CT) and minimum-median CT, inferior–superior ET difference, minimum–maximum ET difference, and standard deviation—offering valuable insights into structural changes over time [52]. ETM can demonstrate diagnostic and monitoring value across a range of OSD. For instance, localized superior thinning of averaged ET was found most in patients with DED (12 out of 21 eyes, Sensitivity/Specificity: 67/88%) when compared to controls, which may be caused by the destruction of limbal stem cells [51]. This postulated mechanism may arise from compensatory increased blinking in response to tear deficiency, resulting in heightened mechanical friction that exacerbates epithelial damage and contributes to the thinning of the superior corneal epithelium. Francoz et al. revealed ET alterations were found to be more pronounced in the peripheral cornea than centrally, with greater changes observed as DED severity increases [53].

In contrast, although corneal thinning (minimal ET ≤ 44 μm) has been reported in DED (AUC 0.88; Sensitivity/Specificity: 86%/47%) [16], Kanellopoulos et al. observed increased ET in DED when multiple stages and etiologies were included, attributing this to compensatory epithelial proliferation. These observations appeared to be device-dependent, likely reflecting differences in tear film incorporation, with the SD-OCT (RTVue-100; Optovue Inc., Fremont, CA, USA) demonstrating greater ET values compared to UHR-AS-OCT, thereby emphasizing the need for careful consideration of imaging modality in both cross-sectional evaluation and longitudinal assessment of OSD [46].

ETM therefore offers a sensitive, non-contact tool for early epithelial remodeling in DED and other OSD. Segmentation of volumetric UHR-AS-OCT scans are now redefined using a joint approach of a super-resolution generative adversarial network that is capable of fine-tuning the varies layers of the cornea, of which en face thickness maps aid in providing a clear overview between the diseased and normal [20]. Its 4 mm field of view demonstrated highest reproducibility (ICC = 0.97) across the whole cornea and stroma, followed by epithelium/Bowman’s complex (ICC = 0.64) and endothelium/Descemet’s membrane complex (ICC = 0.53), respectively. However, the lack of compensation for imaging artifacts (e.g., signal saturation and eyelash shadowing), together with the absence of speckle noise evaluation, may reduce segmentation accuracy and confound the interpretation of thickness-based biomarkers, as observed, which may not accurately represent true tissue changes.

## 4. Advanced Ocular Surface Disease: Structural and Vascular Imaging

Corneal neovascularization (CoNV) can occur secondary to inflammation, infection, trauma, chemical burns, limbal stem cell deficiency (LSCD), or iatrogenically and is one of the leading causes of blindness worldwide requiring corneal transplant [54,55]. While AS-OCT can help to make the distinction between active and regressing or regressed vessels, AS-OCTA provides novel, quantitative, and non-invasive parameters for assessing CoNV severity—severe CoNV revealing increased CoNV posterior limit, thickness, depth percentage, area, and volume as compared to mild CoNV [56]. A semiquantitative assessment can be made by measuring the integrated density of the back shadows produced by the vessels. AS-OCT demonstrated a dense back shadow in all active vessels studied, which was absent in 84.62% at the regressed stage. The intensity of the back shadow was also noted to be reduced in regressing vessels (15.38%) [57].

### Limbal Stem Cell Deficiency

Limbal stem cells, responsible for producing corneal epithelial cells, are crucial for preserving corneal transparency and integrity. Consequently, damage to these cells would lead to disruption of the normal homeostasis of the corneal epithelium, resulting in limbal stem cell deficiency (LSCD). With AS-OCT emerging as a superior tool, it can facilitate both the diagnosis and severity staging of LSCD, which is often unachievable with traditional methods like impression cytology [58]. Earlier, Chan et al. reported a negative correlation between ET and clinical stage of LSCD, underscoring its diagnostic relevance [59]. The staging system for LSCD was introduced based on the extent of conjunctivalization and pannus over the cornea. Stage I was defined as the preservation of central 5 mm cornea—any involvement of this area would be indicative of stage II—while stage III denotes the involvement of the entire surface [60]. In a subsequent study employing SD-OCT (RTVue-100; Optovue Inc., Fremont, CA, USA), they introduced a clinical subscore system (mild: 1–4 points; moderate: 5–7 points; severe: 8–10 points) demonstrating negative correlation only with 3 parameters: basal cell density (BCD) (ρ = −0.628, *p* < 0.001), central epithelial thickness (CET) (ρ = −0.591, *p* = 0.007) and total corneal nerve fiber length (CNFL) (ρ = −0.555, *p* = 0.010), surpassing correlations with limbal measurements [21]. Hence, the adoption of a quantitative grading system incorporating both clinical subscores and objective in vivo biomarkers (e.g., BCD, CET, CNFL) may mitigate the bias associated with reliance on a single parameter and enable a more comprehensive assessment of LSCD in both diseased and normal eyes. LSCD carries the hallmark of conjunctivalization in which this process is associated with chronic corneal inflammation, destruction of basement membrane, pathological vascularization and scarring [45,61,62,63]. The anterior displacement of limbal vessels onto the cornea, thought to occur in response to epithelial thinning, has been postulated as an early clinical marker of LSCD [23]. Hence, assessment of the degree of limbal ischemia is vital in forecasting the likelihood of LSCD and paving early surgical decisions such as tenonplasty.

**Figure 3:**
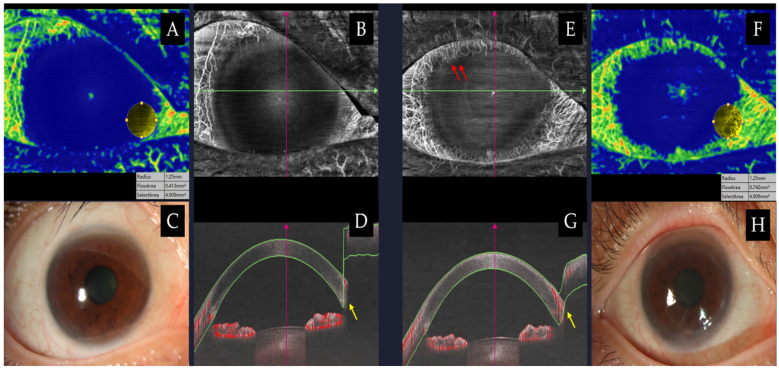
Clinical images of an eye with progressive limbal stem cell deficiency at 6 months (**E**–**H**) vs. baseline (**A**–**D**). (**A**,**F**) CoNV quantification maps demonstrated increased flow area (0.742 mm^2^ at 6 months) as demarcated in yellow and correlates to flow density on cross-sectional scans (yellow arrows). (**B**,**E**) En face maps revealed an increased pannus area extending toward the cornea (red arrows), a detail that may be missed on slit-lamp photography. Original en face images are taken using swept-source AS-OCTA (BMizar, BM-400K, TowardPi Medical, Beijing, China).

Although its etiologies are diverse, chemical ocular injury remains as one of the most common causes of LSCD apart from others such as Stevens–Johnson syndrome and long-term topical medication use. In animal models, alkali concentration-dependent burn intensity biomarkers, in the form of concentration-associated corneal swelling, can be assessed by non-invasive AS-OCT/OCTA, distinguishing between mild, moderate, and severe ocular injury [64]. While conventional slit-lamp examination can identify overt vascularization and epithelial irregularities, it often underestimates subtle or early changes as seen in Figure 3.

Tey et al. revealed that AS-OCTA offers more precise vessel delineation and greater repeatability than conventional imaging in acute-phase rabbit models [65]. This performance was subsequently validated in assessing acute chemical injury in two ways: first, by demonstrating higher interrater agreement for limbal disruption using AS-OCTA compared with slit-lamp examination in affected human eyes (κ = 0.7 vs. 0.4) [66]; second, by using objective AS-OCTA–derived limbal vessel density parameters to distinguish quadrants affected by acute chemical injury from control eyes at presentation. However, this finding was limited to a niche cohort of eyes with acute limbal ischemia (24–48 h), and the authors noted that further studies are needed to validate its applicability across varying severities and grades of chemical injury [66].

**Figure 4:**
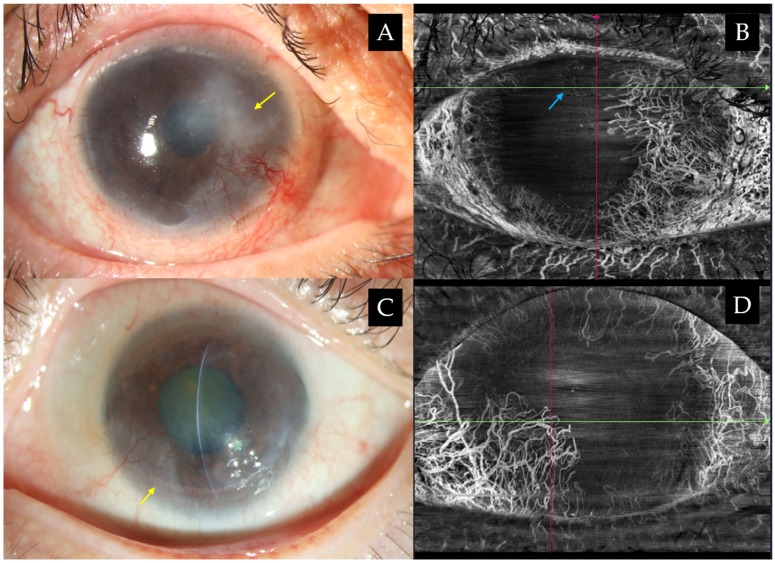
(**A**–**D**) Clinical images of stage II limbal stem cell deficiency illustrating conjunctivalization encroaching the visual axis, resulting in compromised vision. Late stages are often accompanied by subepithelial scarring (yellow arrows) and recurrent or persistent epithelial defects (blue arrow). (**B**,**D**) En-face images demonstrate corneal vascularity in these representative cases and reveal subtle growth that may not be apparent on slit-lamp examination. Original en face images are taken using swept-source AS-OCTA (BMizar, BM-400K, TowardPi Medical, Beijing, China).

AS-OCTA can therefore serve as an adjunctive tool, particularly in eyes with acute chemical burns where FA may pose risks. Serial AS-OCTA scans enable longitudinal follow-up, providing objective assessment of conjunctival vascularization development, which may aid in evaluating subsequent LSCD and its associated complications [23,66]. Fung et al. further demonstrated that AS-OCTA detected a greater extent of limbal ischemia and showed a significant correlation with visual outcomes at 3 months (r = 0.76, *p* = 0.001), suggesting that objective delineation of the extent and depth of limbal conjunctival vascular nonperfusion may enable more accurate prognostication [67]. This was reinforced by Binotti et al. where they established a significant association between disease severity and best-corrected visual acuity (BCVA) with two AS-OCTA metrics: maximum corneal vascular extension (r = 0.547, *p* = 0.001) and corneal vascular thickness (*r* = 0.765, *p* < 0.001), even in early stages of LSCD [23]. Recent evidence also highlights the prognostic potential of AS-OCTA: elevations in deep and superficial vessel diameter indices (VDI) predicted a greater likelihood of corneal melting and keratitis, whereas a decline in deep VDI was significantly associated with an increased risk of conjunctivalization [22]. As such, AS-OCTA could become an important complementary imaging tool for chemical ocular injuries and LSCD, providing objective and reproducible assessments that support accurate diagnosis, grading, and prognosis as seen in Figure 4. These findings highlight AS-OCTA’s potential as a quantitative imaging tool for LSCD severity, correlating vascular indices with clinical outcomes and guiding surgical planning.

## 5. Ocular Surface Squamous Neoplasia: Applications of OCT and OCTA

In healthy eyes, the conjunctiva epithelium predominantly manifests as mildly hyper-reflective layer, with tissue architecture that is less organized in contrary to the more uniform, linear configuration of the underlying subepithelial layer. Ocular surface squamous neoplasia (OSSN) comprises a heterogeneous spectrum of ocular surface growths, ranging from cornea and conjunctiva dysplasia to invasive squamous cell carcinoma, commonly associated with prolonged sun exposure and weakened immune systems. AS-OCT has been employed to distinguish malignant OSSN from benign conjunctival lesions and may reduce the need for histopathology by enabling a non-invasive optical biopsy when characteristic imaging features are identified [68]. Classical features of OSSN include the presence of hyperreflectivity, epithelial thickening and abrupt transition from normal epithelium [38,68,69,70], but these features may not all be present in every case. Vempuluru et al. reported a sensitivity of 93% and a specificity of 70%, attributed to overlap with lesions (e.g., sebaceous carcinoma with squamous differentiation, squamous papilloma, epithelial hyperplasia) for AS-OCT in detecting OSSN [71]. Bejjanki et al. demonstrated the utility of AS-OCT in the evaluation of pigmented OSSN, highlighting characteristic features such as bilateral complexion-associated melanosis and a hyperreflective, thickened epithelium on AS-OCT imaging [72].

Beyond these classic features, AS-OCT can distinguish invasive OSSN from intraepithelial lesions through features such as loss of plane of separation between the epithelium and subepithelial layers and direct visualization of the lesion extending into the subepithelial layer. Additionally, the forms of cystoid spaces can be delineated: type 1 (small, round lesions with hyporeflective compositions) which is indicative of blood vessels, this can be differentiated from type 2 (large, irregular with hyperreflective compositions) as it is indicative of areas of necrosis [70,71]. Karp et al. established the role of UHR-AS-OCT in accurately delineating tumor margins intraoperatively in concordance with histological borders, minimizing healthy tissue removal [73]. Additionally, highest vessel area density (VAD) was observed within conjunctival tumors, followed by adjacent subepithelial tissues, and lowest in tissue located 200 microns underneath the tumors as opposed to the unaffected eye. Distinct morphological and quantitative vascular features like larger vessel diameters and greater perilesional vessel depth are observed in malignant lesions that correspond to feeder vessels.

AS-OCT can facilitate early detection of sub-clinical OSSN, monitor response to topical treatment, and identify early recurrences [74]. Of which, AS-OCTA revealed complete resolution of the tumor in correlation to decrease in subepithelial VAD when treated with topical immunotherapy or chemotherapy, comparable to the fellow non-affected eye [75]. Ghanbari et al. revealed decreases in both superficial VAD (36.3% ± 6.8% at presentation, 33.1% ± 7.5% during treatment, and 30.5% ± 4.9% after tumor resolution, *p*  <  0.05) and VDI (1.87  ±  0.16 at presentation, 1.83  ±  0.13 during treatment, and 1.74  ±  0.1 after tumor resolution, *p*  <  0.05) in OSSN eyes treated with topical interferon α-2b (IFN-α2b); however, they did not observe significant changes in other AS-OCTA parameters [76]. Absolute resolution of OSSN after topical treatment and surgery can be demonstrated by a normal epithelial architecture, with the return to baseline hyperreflectivity and the normalization of epithelial thickness [38,77,78].

In contrast, pterygium is a benign fibrovascular proliferation presenting as a wing-shaped subepithelial lesion extending from the bulbar conjunctiva onto the cornea and may be asymptomatic in its early stages. On AS-OCT, thickness of epithelium may vary but when thickened, epithelial reflectivity can be heterogeneous, hypo- or hyperreflective, with the latter attributed to actinic changes [79,80]. Manifestation of active pathological processes such as fibrosis and inflammatory infiltration can accelerate growth and recurrence [81,82], and once symptomatic or cosmetically evident, the lesion often has encroached upon the corneal optical zone. Thus, timely evaluation and risk assessment are therefore crucial to guide surgical intervention and limit corneal damage. AS-OCT has been utilized to distinguish regressive from progressive pterygium, the latter often demonstrating nodular thickening at the limbus. Progressive disease has shown to associate with greater refractive impact, including increased keratometric astigmatism proportional to the lesion severity [83]. Furthermore, Gasser et al., using high resolution-optical coherence tomography (HR-OCT), reported reduced thickness of the pterygium head and flat bridging of the corneoscleral transition zone, with increased stromal scarring and astigmatism [84].

Detection of features in pterygium recurrence have been reported, namely subepithelial masses with clinically visible margins preoperatively, while residual tissue on both the corneal and conjunctival sides may indicate a higher risk of recurrence [85]. Recently, Niu et al. proposed the combination of AS-OCT parameters and systemic immune-inflammation index (SII) in the monitoring of the condition, with each unit increase in SII representing an increase in the possibility of pterygium progression by 1.01-fold (95% CI: 1.00, 1.01) (*p* = 0.008) [86].

As such, Kieval et al. reported that an ET cutoff of 142 µm, measured using UHR-AS-OCT, could differentiate OSSN from pterygium, achieving a sensitivity of 94% and specificity of 100% [79]. Similarly, Nampei et al. characterized distinct AS-OCTA flow patterns, with “zigzag” vessels in both superficial and deep layers in OSSN, versus predominantly superficial “straight” vessels in pterygium [87]. The use of AS-OCTA has been used to evaluate the healing response of conjunctival autografts in patients undergoing pterygium surgery, clearly delineating the progress of marginal cornea arcades remodeling since failure in reconstruction will likely contribute to the recurrence of pterygium [88]. Karp and colleagues established the relationship of localization of conjunctival tumor margins with the pathologically confirmed margin mark using HR-OCT in all eyes with OSSN [73]. These observations affirm the feasibility of decreasing residual positive margins and minimizing healthy tissue removal through AS-OCT. However, differentiation remains a challenge due to overlapping morphological features as lesion thickness alone is not a reliable indicator of invasiveness [89]. Visualization of the plane in its entirety can be limited by back-shadowing, lesion thickness, intrinsic vascularity, keratin and pigmentation of which a single scan captures only a cross-section of the lesion [71,90]. Despite inherent limitations, AS-OCT and AS-OCTA offer rapid, non-invasive evaluation of lesion extent, anatomical involvement, and treatment response; however, in cases of clinical uncertainty, histopathologic confirmation remains warranted.

## 6. Future Applications

The rapid evolution of AS-OCT and AS-OCTA has transformed imaging of the ocular surface from qualitative assessment to quantitative, reproducible evaluation. Future applications can explore expanding clinical applicability, improving standardization and integrating imaging biomarkers with molecular and functional diagnostics. Advances in UHR-AS-OCT and swept-source platforms allow detailed epithelial, stromal, and tear-film analysis that can be combined with angiographic parameters such as vessel density and flow index. These multimodal datasets could support the development of imaging-defined endotypes for ocular surface disease, including DED, LSCD, and OSSN. Most normative databases for OCT focus on retina and optic nerve head vasculature [91,92]. Establishing normative databases for ET, vessel density, and corneal densitometry would enable standardized staging systems comparable across devices and centers.

AS-OCT and AS-OCTA may serve as objective imaging tools for quantifying structural and vascular changes and for monitoring therapeutic response. While AS-OCT has been used to assess the severity of disorders of CoNV, studies can investigate AS-OCT serving as early potential biomarkers of success after anti-inflammatory, anti-angiogenic, or regenerative therapies. Vascular regression (area, density, depth) on AS-OCTA and epithelial normalization or thickness remodeling on AS-OCT or UHR-AS-OCT are quantitative, reproducible, and sensitive to therapeutic change, supporting their use as early response biomarkers and trial endpoints on the ocular surface. Current deep-learning algorithms integrating with AS-OCT have applications in diagnosing corneal opacities and infective keratitis, but there is yet to be work that investigates the integration of deep-learning algorithms studying vessels that may further enable automated “risk-of-progression” scoring to tailor therapy intensity for patients [93,94].

Emerging precision-medicine approaches seek to correlate imaging signatures with molecular profiles. Tear proteomic and cytokine data acquired using multiplexed platforms can be aligned with AS-OCT/OCTA data to identify molecular correlations of epithelial instability, angiogenesis, and inflammation. This integration may yield composite imaging-molecular biomarkers that stratify patients for targeted therapies, such as photobomodulation, immuno-modulators, or lipid-layer restoration.

In terms of post-operative surveillance, other than using ASOCT/OCTA to investigate recurrence post excision in OSSN, it can also be harnessed to objectively track vascular regression following conjunctival autografting or amniotic membrane transplantation in LSCD, complementing clinical grading and reducing inter-observer variability. Artificial intelligence (AI)-driven AS-OCT/OCTA analytics may facilitate community-level screening for referable OSD. In addition, AI models trained on OCTA-derived vascular features may allow for automated triage and follow-up. Portable OCT platforms coupled with secure cloud-based image interpretation could enable the remote management of chronic OSD and post-surgical surveillance, particularly in low-resource settings.

To support widespread clinical adoption, future work must establish acquisition standards, open reference datasets, and validated segmentation algorithms for anterior-segment imaging. Harmonization of imaging parameters across vendors, along with consensus on reporting standards will accelerate translation into clinical trials and practice guidelines.

## 7. Limitations

This review has its limitations in that it is subject to important methodological heterogeneity across the included studies.

### 7.1. Limitations and Standardization in AS-OCT and AS-OCTA Imaging

Despite the rapid advances in AS-OCT and AS-OCTA, several methodological limitations remain that affect the interpretation and comparability of imaging findings across studies. Standardization of acquisition protocols, reporting parameters, and analysis methods will be essential for the reliable translation of these technologies into routine clinical practice.

### 7.2. Imaging Artifacts

AS-OCTA imaging is susceptible to several artifacts that may influence vascular measurements [95,96]. Common artifacts include motion artifacts [97], which arise from eye movements during image acquisition and may produce vessel duplication or discontinuity; projection artifacts, where signals from superficial vessels are projected onto deeper layers; shadowing artifacts caused by opacities such as corneal scarring or edema; and segmentation errors, particularly in diseased corneas with irregular morphology. These artifacts may lead to over- or under-estimation of vascular density and flow parameters and should be carefully evaluated when interpreting OCTA images.

### 7.3. Acquisition and Reporting Standards

To improve reproducibility and comparability between studies, minimum reporting standards for AS-OCT and AS-OCTA acquisition should be clearly described [98]. Important parameters include scan size and scan pattern, imaging wavelength, axial and lateral resolution, segmentation boundaries used for vascular layer analysis, and the use of motion correction algorithms. In addition, reporting the signal strength or image quality index, averaging methods, and the software used for vascular quantification is essential for the accurate interpretation of quantitative metrics.

### 7.4. Segmentation and Quantitative Analysis

Accurate segmentation of corneal and conjunctival layers remains challenging, particularly in eyes with structural abnormalities. Manual correction or customized segmentation strategies are sometimes required, which introduces potential operator-dependent variability [99].

### 7.5. Cross-Device and Software Variability

Another important limitation is the lack of standardization across imaging platforms. OCT and OCTA systems differ in light source wavelength, scanning speed, signal processing algorithms, and vascular quantification methods [100,101]. Small differences in metrics such as vessel density or flow area may fall within the range of measurement noise rather than representing true biological change. Although OCTA provides high-resolution vascular imaging, repeatability limits and test–retest variability for anterior segment vascular parameters remains incompletely characterized. Future studies should incorporate repeatability analysis and minimal detectable change thresholds to better define the clinical utility of AS-OCTA-derived biomarkers. Consequently, quantitative metrics such as vessel density, flow area, or perfusion indices may vary significantly between devices and software versions. Direct comparison of numerical values across different platforms should therefore be approached with caution unless cross-platform validation studies have been performed [100].

### 7.6. Need for Reproducibility Studies

Before AS-OCTA metrics can be widely adopted as clinical biomarkers, further work is required to establish repeatability and test–retest variability for anterior segment vascular parameters. Quantitative metrics are derived from different devices (e.g., RTVue, Avanti, Casia2, BMizar), with varying axial resolutions, scan protocols, segmentation algorithms, and study populations, thereby limiting direct numerical comparability. Although UHR-AS-OCT achieves axial resolutions <5 μm (and in some cases ~1–2 μm), superior technical resolution does not necessarily translate into proportional gains in diagnostic accuracy, sensitivity, specificity, or longitudinal reproducibility. Few head-to-head comparisons with conventional systems (5–25 μm) are available, and resolution should not be interpreted as a surrogate for clinical superiority. Reported TFT values vary widely (3–46 μm), reflecting differences in optical principles and anatomical interfaces measured. OCT-derived TFT likely captures a subset of tear film structures rather than total thickness, and absolute values are therefore not interchangeable across modalities. Establishing consensus acquisition protocols and standardized reporting guidelines will be crucial for enabling multi-center studies and facilitating clinical translation.

## 8. Conclusions

AS-OCT and AS-OCTA are primed to become central tools in precision ocular surface medicine, providing objective, quantitative, and reproducible biomarkers that bridge imaging, molecular data, and patient outcomes. Integration with AI analytics will enable real-time, individualized monitoring, ultimately improving diagnostic accuracy, therapeutic decision-making, and visual outcomes. As AS-OCT and AS-OCTA technologies continue to evolve, their integration into routine ocular surface evaluation will hinge on standardized acquisition protocols, the automated segmentation algorithms, and large-scale validation. Therefore, future work should emphasize multi-center trials and correlation with molecular and proteomic biomarkers to enable precision diagnosis and monitoring of ocular surface disease.

## Figures and Tables

**Table 1:** Evidence appraisal of proposed OCT/OCTA biomarkers in ocular surface diseases.

Structure/ Disease	Instrumentation	Commercial Availability	Evidence Level	Reproducibility	Potential Applications	Key Limitation/Artifact
Dry Eye [17]	Custom-built spectral domain UHR-AS-OCT (axial resolution of ~3 µm)	Limited	Prospective, Interventional Case–control Study	Good reproducibility (ICC: 0.94) (n = 8 eyes) Minimal operator dependency (ICC: 0.96) (n = 5 eyes)	Higher EIF than controls (5.79 vs. 0.77, *p* < 0.001) correlated to subjective tool (*r* = 0.778, *p* < 0.001)	Time consuming in calculation of EIF
Lipid Layer (Tear Film) [18]	Custom-built spectral domain UHR-AS-OCT (axial resolution of 1.2 µm)	Limited	Retrospective	Good interrater reliability (ICC: 0.89)	Qualitative TFLL pattern: Dotted (DOT): a dark background with bright spots. Most stable type of TFLL.	Operator dependent; LLT values and TFLL pattern images are not acquired exactly at the same time.
Tear Film [19]	Custom-built spectral domain UHR-AS-OCT (axial resolution of 1.38 µm)	Non-commercial	Prospective Cross-sectional Observational study	Not reported	Rapid rate of PCTF thinning in asymptomatic and symptomatic MGD; Slower rate of PCTF thinning associated with thicker lipid layers.	Limited integration of other ocular surface tests Physiological variability of tear film Non-commercial prototype No reproducibility analysis
Cornea Epithelium [20]	Custom-built spectral domain UHR-AS-OCT (axial resolution of <2 µm)	Non-commercial	Retrospective Methodological study	Entire cornea and stroma (ICC = 0.97) Epithelium/Bowman’s complex (ICC = 0.64) Endothelium/Descemet’s membrane complex (ICC = 0.53)	Segmentation of volumetric UHR-AS-OCT coupled with super-resolution generative adversarial network (SRGAN) fine-tune layers of the cornea with good reproducibility.	Lack of compensation for imaging artifacts (e.g., signal saturation and eyelash shadowing); Absence of speckle noise evaluation (may reduce segmentation accuracy).
Corneal Neovascularization [21]	Spectral domain AS-OCT (RTVue-100; Optovue Inc., Fremont, CA, USA)	Commercial	Observational Cross-sectional Comparative Study	Inter-observer variation < 5% for most parameters (BCD, CET, ET)	Clinical subscore system for LSCD based on the central cornea with strong negative correlation with central epithelial thickness. Mild: 1–4 points Moderate: 5–7 points Severe: 8–10 points	Lack of full reproducibility analysis; Clinical utility of these biomarkers requires further validation in larger multicenter studies; Evaluation of cell morphology is relatively more subjective than the assessments of the other parameters and depends on the experience of observers.
Corneal Neovascularization [22]	Spectral domain AS-OCT (RTVue-100; Optovue Inc., Fremont, CA, USA)	Commercial	Prospective Observational study	Not reported	Elevations in deep VDI predicted a greater likelihood of corneal melting and keratitis; Decline in deep VDI was significantly associated with an increased risk of conjunctivalization.	Small sample size; Single-session baseline measurement; Single center.
Corneal Neovascularization [23]	Spectral domain AS-OCTA (Avanti XR AngioVue, Optovue, Inc., Fremont, CA, USA)	Commercial	Retrospective, Cross-sectional, Case–control Study	Excellent intragrader repeatability and inter-grader reproducibility (all ICCs > 0.900, *p* ≤ 0.001)	Significant difference in CoVE and CoVT between all stages compared to controls, and between stage I and III LSCD (*p* < 0.001); BCVA showed strong correlation with CoVT (*r* = 0.765, *p* < 0.001) and moderate correlation with CoVE (*r* = 0.547, *p* = 0.001).	Retrospective design; Relatively small number of subjects per group.
Cornea Epithelium [24]	Spectral domain AS-OCT (RTVue-100; Optovue Inc., Fremont, CA, USA)	Commercial	Retrospective Comparative Study	Device dependent	LSCD: spoke-wheel pattern (max-min ET: ≥14 μm); EBMD: inferior thickening pattern (central ET: >56 μm); Dry eye: superior thinning pattern (minimal ET < 44 μm).	Severity of diseases was not graded; Tear film discrimination inability.
Ocular Surface Squamous Neoplasia (OSSN) [25]	Spectral domain AS-OCT (RTVue-100; Optovue Inc., Fremont, CA, USA)	Commercial	Methodological	Device dependent	Detection of sub-clinical OSSN (thickened and hyperreflective epithelium with an abrupt transition point) for predicting histologic tumor margins.	No comparison with gold-standard diagnostics; No formal comparison with histopathology.

UHR-AS-OCT—ultra-high-resolution anterior segment optical coherence tomography; AS-OCT—anterior segment optical coherence tomography; ICC—intraclass correlation coefficients; EIF—epithelial irregularity factor; TFLL—tear film lipid layer, LLT—lipid layer thickness; PCTF—pre-corneal tear film; MGD—meibomian gland dysfunction; BCD—basal cell density; CET—central epithelial thickness; ET—epithelial thickness; LSCD—limbal stem cell deficiency; VDI—vessel diameter indices; CoVE—corneal vascular extension and CoVT—corneal vascular thickness; EBMD—epithelial basement membrane dystrophy.

## Data Availability

The original contributions presented in this study are included in the article. Further inquiries can be directed to the corresponding author.
